# Research Progress of Bioinspired Nanostructured Systems for the Treatment of Ocular Disorders

**DOI:** 10.3390/ph16010096

**Published:** 2023-01-10

**Authors:** Xuan Chen, Rui Yang, Jinyan Shen, Qingyu Huang, Zhifeng Wu

**Affiliations:** 1 Department of Ophthalmology, Wuxi Second People’s Hospital, Nanjing Medical University, Wuxi 214002, China; 2 Research Institute for Reproductive Health and Genetic Diseases, Wuxi Maternity and Child Health Care Hospital, Wuxi School of Medicine, Jiangnan University, Wuxi 214002, China; 3 Department of Ophthalmology, Affiliated Wuxi Clinical College of Nantong University, Wuxi 214002, China

**Keywords:** bioinspired, nano, anterior segment, posterior segment, ocular disorders

## Abstract

How to enhance the bioavailability and prolong the residence time of drugs in the eye present the major barriers to traditional eye delivery. Nanotechnology has been widely used in ocular drug delivery systems because of its advantages of minimizing adverse reactions, decreasing the frequency of administration, prolonging the release time, and improving the bioavailability of the drug in the eye. As natural product-based nanostructured systems, bioinspired nanostructured systems have presented as less toxic, easy to prepare, and cost-effective and have potential application value in the field of nanotechnology. A systematic classification of bioinspired nanostructured systems based on their inspiration source and formulation and their brief applications in disease are presented here. A review of recent research progress of the bioinspired nanostructured systems for the treatment of the anterior and posterior segment of ocular disorders is then presented in detail. Finally, current challenges and future directions with regard to manufacturing bioinspired nanomaterials are provided.

## 1. Introduction

Anatomically, the eye can be divided into the anterior and posterior segment. The anterior segment is the ocular tissues anterior to the lens, mainly including the cornea, conjunctiva, iris, ciliary body, anterior portion of the sclera, and the lens itself. Inflammation, injury, and cataract are the major ocular disorders that occur in the anterior segment. Clinically, traditional ocular dosage forms, such as eye drops and eye ointments, are commonly used to treat local ocular disease of the anterior segment. Yet, as reviewed by Awwad et al., due to the physical and biochemical barriers of eye including the pre-corneal tear film, the structure and biophysical properties of the cornea, reflex tear, tear drainage system, or limited capacity of aqueous humor outflow, limited bioavailability has been recorded for the conventional ocular dosage forms [1]. The posterior segment of eye is the tissues behind to the lens, which comprises the vitreous body, retina, choroid, posterior portion of the sclera, and optic nerve. Posterior segment eye diseases include glaucoma, age-related macular degeneration (AMD), macular edema (ME) secondary to retinal vein occlusion (RVO), cytomegalovirus (CMV) retinitis, posterior uveitis, uveitis diabetic retinopathy (DR), and retinitis pigmentosa. Delivery of the drug to the posterior segment is challenging. Intravitreal injection (IVI) is the main administration method for the therapy of posterior segment diseases, such as AMD and DR [2]. Although IVI can achieve therapeutic drug concentrations, it may result in infection, hemorrhage, cataracts, and retinal detachment [3]. Due to its rapid elimination, the drugs are usually administered as multiple injections, which has been shown to further increase the risk of side-effects [4]. To address these problems of traditional ocular dosage forms, there is an urgent need to develop new and more effective ocular drug delivery systems.

In recent years, nano-based materials for drug delivery applications have been extensively and deeply explored [5,6,7]. Nano-based materials for ocular drug delivery can not only controlled and sustain release drugs, but also prolong drug retention time, showing promising results. Afarid et al. summarized the commonly used nano-based materials for ocular drug delivery, including magnetic-based materials (iron oxide-based materials, gold-based materials, silica-based materials) and polymeric-based materials (lipid-based materials and polysaccharide-ride-based materials), for the treatment of ocular diseases [8]. It was also mentioned by Afarid and Yang that despite the unique advantages of nano-based materials for ocular drug delivery like small size, controlled release, better solubility, biocompatibility, high bioavailability, and good penetration of ocular barriers, their side effects, especially those caused by inorganic nanoparticles such as genotoxicity, intraocular inflammation, and corneal cell apoptosis, must be considered [8,9]. Bioinspired nanostructured systems, which are synthesized by biological materials or directly mimic the techniques/processes that exist naturally in plants, microorganisms, or animals, are considered to have wide range of sources, better biocompatibility, and less side effects [10]. To the best of our knowledge, no previous reviews detail the progress of bioinspired nanostructured systems for the treatment of ocular disorders. The current review tries to provide a systematic classification of biomimetic nanomaterials based on their inspiration source and formulation. A review of the applications of bioinspired nanostructured systems in the therapy of the anterior and posterior segment ocular disorders is presented in detail. The issues concerning the current challenges and future directions are also raised and discussed.

## 2. The Classification of Bioinspired Nanostructured Systems

**Figure 1:**
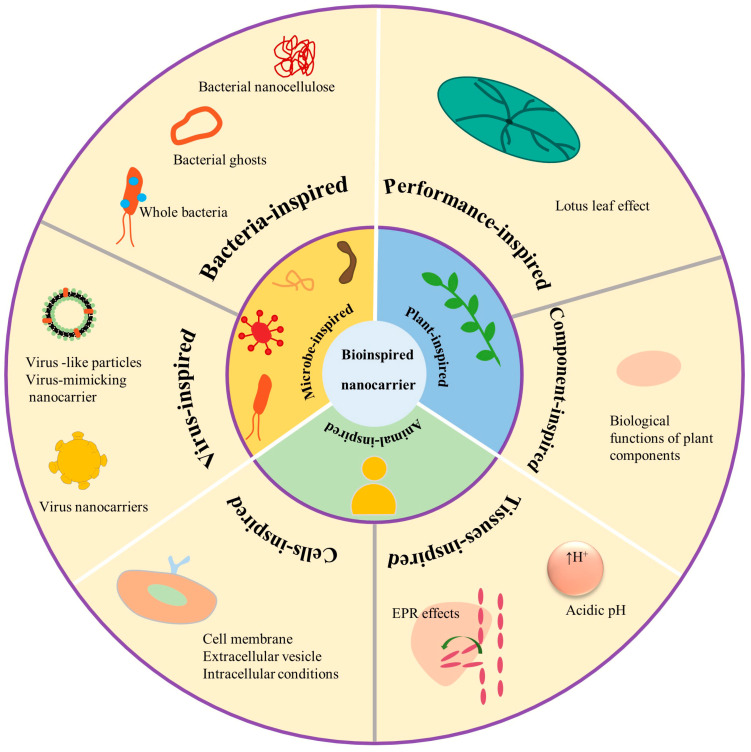
Schematic of a bioinspired carrier developed by the source and formulation of representative life from three kingdoms including microbe, plant, and animal. Abbreviations: EPR, enhanced permeability and retention.

With the advancements in nanotechnology, the synthesis methods of nano-drug delivery systems have been rapidly developed. Bioinspired nanostructured systems been extensively exploited in the pharmaceutical field due to their properties of low toxicity, treatment effectiveness, and target ability. Based on the inspiration source and formulation, these bioinspired nanostructured systems were defined into three classes including microbe-inspired, plants-inspired, animal-inspired drug delivery systems (Figure 1). Inspired by biological functions and developed by mimicking their performance (shape, structure, appearance, locomotion, and surface) or utilizing their components to achieve their biological functions with the help of nanotechnology, bioinspired nanostructured systems have been a newer generation drug delivery system [10,11,12]. Since a large number of studies have been performed and we might not be able to grasp it comprehensively, we will focus on a few specific questions.

### 2.1. Microbe-Inspired Drug Delivery Systems

Due to the unique properties including biodegradability, low toxicity, biocompatibility, strong penetration, and tissue colonization ability, microbe-inspired drug delivery systems are widely used in the therapy of diseases such as cancers, inflammation, and others. Bacteria and viruses are the major source of drug delivery systems. Below we focus on the most common nanostructured systems including bacteria-inspired and viruses-inspired drug delivery systems [10].

#### 2.1.1. Bacteria-Inspired Drug Delivery Systems

Bacteria is the usual microbe. The materials of bacteria-inspired drug delivery systems are derived from the whole bacteria, their derivatives, or secretions. Advances in nanotechnology and biomedical engineering allows bacteria to be an effective carrier to deliver drugs to treat several diseases such as metabolic disease, inflammation, and cancer through physically, chemically, or biochemical modifying [13,14].

In the cases of cancer therapy, the whole bacteria applied for bacteria-inspired drug delivery systems is partly live attenuated or inactivated bacteria. According to the unique characteristics of chemotaxis and colonization, the whole bacteria can be constructed for treating for different types of cancers. The facultative or obligate anaerobic bacteria, such as *Salmonella*, *Escherichia coli*, *Clostridium*, and *Bifidobacterium*, has the advantage of tissue penetration ability, selective colonizing, or proliferative ability in hypoxic regions, which can deliver the therapeutics to the core of the solid tumor (Table 1). Inspired by the photosynthesis of *S. elongatus*, Wang et al. [15] utilized the upconversion nanoparticles (UCNPs) to convert near-infrared excitation into visible light to activate S. elongatus in a preclinical stroke animal model. The results showed that the S. elongatus can generate oxygen, promote neovascularization, and improve neuronal function recovery, thus treat the ischemic stroke. However, the likelihood of a mutation occurring in some bacteria may result in the unintended side effects for the host. As outer membrane vesicles, bacterial ghosts (BGs) derived in bacteria still preserve the target ability and immune-modulatory function of the living bacteria while avoiding infection; they have been developed as nanocarriers against tumors, inflammation, and infection, among others [16]. Go et al. [17] constructed the *Escherichia coli* extracellular vesicle-mimetic ghost nanovesicles (ghost NVs) as biological nanocarriers to deliver anti-inflammatory drugs to human U937 monocytes. The ghost NVs can mitigate the symptoms of systemic inflammatory response syndrome in the mice, which suggests a potential application to deliver drugs for therapy of diseases such as bacterial sepsis. As the secretion of bacteria, bacterial nanocellulose (BNC), also called bacterial cellulose (BC), is widely derived from several species of *Gluconacetobacter* including *Gluconacetobacter xylinus*, *Sarcina,* and *Komagateibacter*. BNC has the advantages of a large surface area, high porosity, ease of sterilization, excellent tensile strength, and good biocompatibility, and has been used in tissue engineering, skin tissue repair, and wound healing. Zikmundova et al. [18] reported the BNC could not only enhance the solubility and the penetration of the anti-bacterial and healing agent curcumin when prepared at a high temperature, it could also reduce the cytotoxic of curcumin for human dermal fibroblasts and enhanced the antibacterial activity. Not only that, studies have shown that the microenvironment is not limited to a low pH of the bacterial infection lesion and biofilm makes it possible to design a smart targeted and responsive nanocarrier for antimicrobial therapy [19,20]. Also, inspired by the corkscrew motion of bacteria flagella, nanorobots has received great attention in the field of drug delivery [21,22].

#### 2.1.2. Virus-Inspired Drug Delivery Systems

Viruses are smaller in size than bacteria. Thus far, viruses have a wide range of biomedicine applications including vaccination, gene therapy, and target drug delivery. Virus-inspired drug delivery systems have been extensively studied. Within this section, we focused on three important kinds including virus nanocarriers, virus-like particles (VLPs), and virus-mimicking nanocarriers.

Since viruses can evade most of the host immune system and delivery its genetic material in the host cell, they have been considered natural carriers for drugs, proteins, and nucleic acids. The architecture of virus nanocarriers consists of protein cages and viruses. Virus nanocarriers isolated from bacteria and plant are not only biocompatible and biodegradable, but also non-infectious and harmless to humans. An analysis of published data by Ojha et al. [31] showed that some plant virus nanocarriers including Cowpea Mosaic Virus (CPMV), Red Clover Necrotic Mosaic Virus (RCNMV), and Tobacco Mosaic Virus (TMV) have been used to specifically deliver the anticancer drugs and bioimaging agents to tumors. Velázquez et al. [32] reported for the first time that the turnip mosaic virus (TuMV), naturally infecting cruciferous plants but not humans, can be functionalized by epigallocatechin gallate and showed a significant capacity of tumor homing and cell internalization compared with free epigallocatechin gallate. Furthermore, epigallocatechin gallate functionalized TuMV nanoparticles showed a stronger antibacterial effect on *Sarcina lutea*, *Pseudomonas aeruginosa*, and *Dickeya dadantii* compared with epigallocatechin gallate, suggesting the potential of TuMV as antimicrobial agent nanocarriers [33]. Bacteriophages, also called phages, are viruses of bacteria and are extensively used to load drugs or proteins for the diagnosis and treatment of microorganisms due to their safety profile and flexible genetic engineering property [34]. In addition to functioning as nanocarriers, an abundant carbon and nitrogen source might make bacteriophages as an alternative material for fluorescent nanoparticles. In a recent study, Lai et al. [35] have firstly used the M13 bacteriophage as a carbon source to synthesize carbon-based fluorescent nanoparticles through a one-step hydrothermal carbonization method.

VLPs are the nanoscale structures (0.1–100 nm) consisting of a viral protein and lack of viral nucleic acid and are thus non-infectious. Up to date, VLPs have been produced by various organisms, including bacteria, plants, mammals, and insects. The cavity of the VLPs interior, which can load peptides, protein, drugs, genes, or tracers, makes it an efficient delivery system for biological applications [36]. Moreover, VLPs can be also self-assembled in vitro and this kind of VLP cannot only avoid encapsulation of host-derived components, it also makes the drug loading more convenient compared with in vivo assembly [37]. Tan et al. [38] display a short peptide (GSRSHHHHHH) at the C-terminal end of turnip yellow mosaic virus coat protein (TYMVc) to produce a recombinant protein named TYMVcHis6, which can be self-assembled into robust and thermal stable VLPs. The capacity of the chimeric VLPs to self-assemble after chemical denaturation suggests their potential application as a nanocarrier for target therapy.

As well as taking advantage of live viruses and VLPs, inspired by the functional domains of viral components, virus-mimicking nanocarriers have been widely investigated. The localization in tissues remains a key barrier in the eradication of HIV-1. Studies showed that cluster of differentiation (CD)169 expression on the surface of Siglec1 macrophages can recognize and bind to monosialodihexosylganglioside (GM3) in the HIV-1 membrane to facilitate the virus propagation [39,40]. Inspired by HIV-1, Eshaghi et al. [41] have developed GM3-presenting, two antiretrovirals (ARVs), rilpivirine (RPV), and cabotegravir (CAB)-loaded, lipid-coated polylactic acid (PLA) and poly (lactic-co-glycolic acid) (PLGA) nanoparticles. The results showed that the virus-mimicking nanoparticles could not only target Siglec1 macrophages but also achieve long-inhibition lasting up to 35 days.

### 2.2. Plant-Inspired Drug Delivery Systems

Within this section, we focused on two important kinds of plant-inspired drug delivery systems developed by mimicking the plant’s performance and utilizing their components to achieve their biological functions with the help of nanotechnology. The micro/nanostructure of plants confers many special functions and properties to themselves. Plant-inspired microstructure drug delivery systems have been studied. Lotus leaf effect is a superhydrophobic phenomenon, which is mainly caused by the nano/micro scale rough structures of the lotus leaf that make droplets remain spherical on the surface. Inspired by this, Song et al. [42] transferred the aqueous droplets containing a mixture of a sodium alginate solution, the dilution of calcium chloride solution, and drug onto the superhydrophobic substrate to prepare the drug-embedded calcium alginate (Ca-Alg) hydrogel particles. The process is easy to control and the Ca-Alg particles exhibited a high encapsulation efficacy of acetaminophen of more than 88% and a pH-responsive releasing property. However, fewer studies have been performed on the plant structure-inspired nanostructure drug delivery system. A recent study showed that the biological functions of plant components have potential in preparing the therapeutic nanocarriers. The gallol moieties, which are widely found in antioxidative plant polyphenols, showed a characteristic of low critical micelle concentration (CMC) and the ability to resist enzyme degradation. Inspired by this, a drug delivery system called plant-inspired Pluronic-gallol micelles was designed by Kim et al. to protect micelles from enzymatic and physical degradation through the incorporation of gallol moieties [43]. The results showed that the plant-inspired Pluronic-gallol micelles with a low CMC (10 μM) provide comparatively higher stability than the polymer alone. Coupled with their high cytocompatibility (~97%), the stable micellar systems may be a promising multifunctional vehicle for drug delivery. Recent studies showed that plant-derived components and exosome-like nano-vesicles has the potential to be therapeutic drug or nanocarriers [44,45,46]. As these drug delivery systems are not within the scope of this paper, they will not be further discussed here.

### 2.3. Animal-Inspired Drug Delivery Systems

The kinds and activities of animals are both diverse and complex and have offered a rich inspiration for the design of nanocarriers. Recent years have seen an enormous progression in the field of animal-inspired drug delivery systems [47,48]. In this part, we mainly discuss the cell- and tissue-inspired drug delivery systems and their applications from simple to highly complex biological forms.

#### 2.3.1. Cell-Inspired Drug Delivery Systems

Cells are the basic units of the body responsible for the complex activities including survival and specific functions. As one kind of cell-inspired delivery systems, the cell membrane, including the whole-cell membrane, nanocarrier coated membrane, and cell extracellular vesicle-inspired drug delivery systems are presented first. This section also covers ligand-receptor interaction-inspired active target drug delivery systems. In the final part, we also offer an overview of the intracellular lower pH, reduction–oxidation, and transmembrane potential-inspired stimuli-responsive nanoparticles [49,50,51].

Inspired by cells features, cell-based information exchange, and interpersonal communication, a variety of nanocarriers deriving from the whole-cell membrane including red blood cell, platelet, macrophage, monocyte, neutrophil, stem cell, T cell, natural killer (NK) cell, and adipocyte have been developed [52,53,54]. In what follows, we will take the red blood cells as an example to present and discuss the advancements. A broad diversity of developed nanocarriers is still facing challenges such as stability and clearance in circulation of in vivo delivery. Red blood cell hitchhiking (RH), a new drug delivery platform developed through the combination of nanocarrier and red blood cells, has dramatically changed the behavior of nanocarriers in vivo and has been widely used in the treatment of disease [55]. CD47, which is expressed on the surface of the red blood cells, can send a “do not eat me” signal through binding with signal regulatory protein alpha (SIRPα) on macrophages, and thereby present a long cycle life [56,57]. Ding et al. [58] prepared the RH through adsorbing the methylprednisolone sodium succinate (MPSS) loaded chitosan nanoparticles on the surface of red blood cells (RBC-MPSS-CSNPs) via an electrostatic interaction to relieve the side effects caused by long-term administration of high doses of glucocorticoids. The results showed that the RBC-MPSS-CSNPs can significantly prolong circulation times and alleviate lung injury mediated by lipopolysaccharide in rats. The functions of the cell are mainly governed by the distinct membrane; hence, the cell-membrane has been directly used as raw materials to synthesize nano-formulations. The approach to extract natural cell membrane structures for nanoparticle design can be broadly categorized into two types, one is through human interventions such as serial sonication, electric pulses, or extrusion, and the other is biological extracellular vesicles that are directly secreted by cells under normal, physiological, or pathological conditions. The first such cell membrane-derived nanomaterials are extracted from the red blood cells through a membrane extrusion process [59], which showed a superior circulation half-life in mice. Membrane-enclosed sacculi produced by cells known as extracellular vehicles (EVs), which carry protein, DNA, and RNA cargo of the parent cell [53]. Red blood cell extracellular vesicles (RBCEVs) have a great advantage as nanocarriers because of their non-tumorigenicity, low immunogenicity, easy accessibility, and capability for expansion [60]. Peng et al. [61] reported that the RBCEVs isolated from healthy people through ultracentrifugation were used to load and deliver 5′ triphosphorylated RNA molecules as RIG-I agonists for anti-cancer immunotherapy. The results showed that RBCEVs could deliver RIG-I agonists to the tumor site and metastatic lesions to perform immunotherapy in mice.

The receptors are special proteins existing on the cell-surface and intracellular, which can recognize and bind with extracellular ligands or signaling molecules and thereby responding to external stimuli. Inspired by receptor ligand binding, receptors overexpressed in some disease state provide a more effective drug delivery manner for active nanocarriers modified by the ligands. These nanocarriers modified by ligands, including antibodies and their molecular fragments [62], small molecules (e.g., folate [63] and galactose [64]), lectin [65], and peptides [66], result in a single target ability for some cells. For example, CD44 is a cell surface glycoprotein overexpressed highly expressed in ovarian, pancreatic, breast, and lung cancer. The target nanocarrier modified by the variety of different CD44 ligands including the CD44 antibodies, peptide, hyaluronic acid, chitosan, and chondroitin sulfate have been established for the therapy of tumors [67]. In our previous studies, the CD44 peptide modified polymer micelles [68] and chitosan functionalized liposomes [69,70] were successfully synthesized and have shown an excellent in vivo and in vitro anti-tumor effect compared with the non-target nanocarriers.

The intracellular pH, redox potential gradients, and transmembrane potentials under pathological conditions are different from normal physiological conditions. The most comprehensive example of this is tumor cells. Here we will focus on the stimuli-responsive nanoparticles inspired by tumor intracellular microenvironmental changes. The pH value in tumor cellular endo/lysosomes is even lower (pH 4.0–6.0) than normal tissues (pH 7.4) [51]. The tertiary amino groups (pKa ~6.5) in the interior of the polyamidation (PAMAM) dendrimers can be protonated under slightly acidic conditions, imparting their pH-responsive characteristics. Zhang et al. [71] grafted hydrophobic moieties 4-diethylaminophenyl isothiocyanate (DAITC) onto PAMAM polymers to prepare a nanocarrier that is stable at normal pH and can break down and release protein in acidic conditions in tumor cells, such as in mouse breast carcinoma cells (4T1), human cervical cancer cells (HeLa), and human hepatoma cells (HepG2). In addition, inspired by the redox potential gradients in tumor cells, disulfide bonds were introduced into nanocarriers to realize the drug release through triggering cleavage of the disulfide bond under high concentrations of glutathione [49]. Tumor cells present significantly increased transmembrane potentials, and the triphenylphosphine (TPP) used in delocalized lipophilic cations is often used to modify the nanocarrier for the mitochondrial targeting [49,50].

#### 2.3.2. Tissues-Inspired Drug Delivery Systems

Nanocarriers can selectively accumulate at the focal lesion based on the different characteristics between physiological and pathological conditions. The typical example is the enhanced permeability and retention (EPR) effect-based nanoparticles preferential accumulation in tumor tissues, which was firstly reported in 1986 [72]. In subsequent studies, similar EPR effects were also found in atherosclerosis [73] and myocardial infarction [74]. In particular, nanoparticle size affects the EPR effect in different tissues. Nanoparticles with a diameter of 100–200 nm show a high accumulation efficiency in many solid tumors, while nanoparticles with a diameter less than 100 nm appear to be more often enriched in atherosclerosis tissue [73]. Furthermore, acidic pH is the unique microenvironment of bone resorption lacuna (pH 3–4) [75] and tumor tissue (~6.5) [76], which inspired many research groups to develop different pH-responsive nanocarriers such as pH-responsive metal-organic frameworks (MOFs), pH-responsive liposomes, pH-responsive polymeric micelles, and pH-responsive dendrimers.

## 3. The Classification of Bioinspired Nanostructured Systems

**Figure 2:**
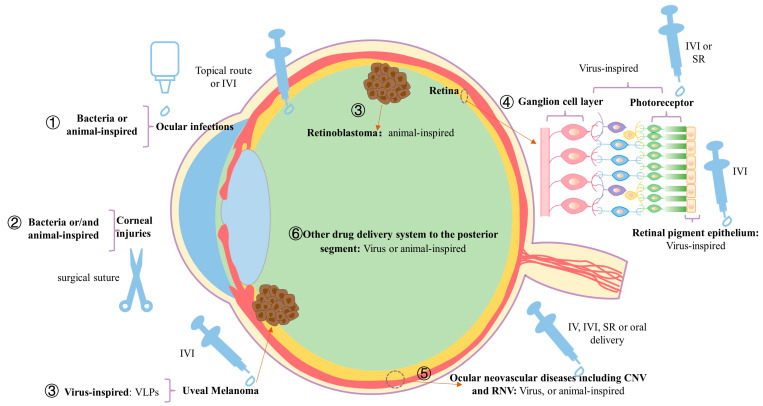
**Applications of the bioinspired nanocarrier for ocular disorders:** (1) Ocular infections: the inspirations include the acid environment produced by the bacteria, the temperature of humans, and the receptors expressed on the surface of the corneal epithelial cells. (2) Regenerative ophthalmology: the inspirations include stem cell-derived EVs, BNC, and BNC-loaded human stem cell-derived cells. (3) Eye tumors: the inspirations include the VLPs and the receptors expressed on the surface of tumor cells. (4) Retina-targeting gene therapy: the inspirations include the virus itself. (5) Ocular neovascular disease: the inspirations include the virus, cell membrane, and EVs. (6) Other drug delivery systems to the posterior eye: the inspirations include the VLPs, the special environment of the ocular disorders, and the receptors expressed on the surface of corneal epithelial. Abbreviations: IVI, intravitreal injection; VLP, virus-like particle; SR, subretinal; IV, intravenous; CNV, choroidal neovascularization; RNV, retinal neovascularization; EVs, extracellular vesicles; BNC, bacterial nanocellulose.

Limited by the local barriers of eyes, including the tear film, conjunctival barrier, blood-aqueous, and blood-retinal barrier, the traditional formulations of ocular drugs are facing challenges of inefficient targeting, low bioavailability, short retention, and inadequate tissue penetration [8]. Mofidfar et al. [77] reviewed, in detail, the nanocarriers used for anterior segment ocular drug delivery, which included liposomes, niosomes, solid lipid nanoparticles (SLNs), nanostructured lipid carriers (NLCs), inorganic nanoparticles, polymeric micelles, nanosuspensions, hydrogels, and nanoemulsion. Therein, liposomes, nanosuspensions, hydrogel, NLCs, and SLNs have been shown to deliver in a sustainable manner, and enhance the accumulation and bioavailability of drugs. Through conclusive shreds of evidence, Wang et al. [78] summarized that many nanotechnology-based drug delivery systems including liposomes, polymeric nanoparticles, nanoemulsions, polymeric nanomicelles, dendrimers, inorganic nanoparticles, supramolecular nanoparticles, double hydroxides (LDH) nanocomposites, star-shaped polymers, and layered, etc., have been used for posterior disorders. However, further risk to human health may arise from the accumulation of nanocarriers in the body and the inefficient safety evaluation. In some cases, ordinary nanocarriers may present as toxic to the eye tissues [79]. Developing nanocarriers using natural eco-friendly material is an important approach to reduce toxicity. To this purpose, the bioinspired nanocarrier, a more human body-friendly nanostructure, which is inspired by nature’s own designs and solutions and has great potential for biomedical applications, has been used in ocular disorders. As far as we know, there have been no comprehensive systematic reviews about the bioinspired nanostructured systems for the treatment of ocular disorders. Here, we try to provide an overview of applications of the bioinspired nanocarrier for ocular disorders. We reviewed the positive investigations so far and summarized six core applications (Figure 2).

### 3.1. Bioinspired Nanostructured Systems for the Treatment of Anterior Segment Ocular Disorders

The applications of bioinspired nanocarriers for anterior segment ocular disorders include, but are not limited to, the following ocular infections and regenerative ophthalmology. As shown in Table 2, owing to space limitations, we will take some representative investigations as examples.

Ocular infections, including keratitis and endophthalmitis, refer to the local tissue inflammatory response caused by the invasion of bacteria, viruses, or other microbes. Keratitis is a leading cause of visual impairment and blindness. Due to increased contact lens use or corneal surgery, the incidence of keratitis has been increasing in recent years [88,89]. Although nanocarrier-based delivery systems can eliminate or control the infection, some problems including non-specific and uncontrolled release still exist. Therefore, development of nanocarrier with the ability of controlled and targeted drug release is very important. Inspired by selective interactions set between the corneal cell receptors and specific ligands, an active target delivery system has been developed. Ahsan et al. [83] prepared anti-toll-like receptor (TLR4) antibody-modified nanoparticles to target the TLR4, which is highly expressed on the corneal epithelial cells when infected to treat keratitis. The results showed that the nanoparticles can significantly increase corneal retention, suppress inflammation, and resolve the infection in a rat model of keratitis. To achieve controlled drug release, various endogenous stimuli (acid environment, ion, enzyme, temperature, H_2_O_2_)-responsive drug delivery systems have been developed for the therapy of ocular diseases. The thermosensitive characteristic of Pluronic F127 (PF127) has been used to engineer the in-situ gel for ophthalmic disease [90]. However, rapid loss of drugs caused by eye irritation at high concentrations and unsatisfactory mechanical strength is the main barrier for the application of PF127 in eye disease [91,92]. The hydroxy propylmethylcellulose, which can enhance the thermal and mechanical properties of PF127, has been introduced by Tavakoli et al. [85] to synthesize an in situ thermosensitive gel containing nanoparticles. The results showed that the contact time of drug-corneal has been prolonged by the optimized gel compared with pristine gel. Endophthalmitis can not only arise in the anterior segment but also in posterior segments. Inspired by the acidic microenvironments produced by bacteria, a new composite nanomaterial comprised of pH-responsive zeolitic imidazolate framework-8-polyacrylic acid (ZIF-8-PAA) and polyacrylic acid was recently designed by Chen et al. [81] for anti-bacterial infections and to eradicate biofilm in endophthalmitis. This pH-responsive nanomaterial showed good therapeutic propertied in a mouse endophthalmitis model.

Regenerative medicine for the cornea, as an alternative to traditional keratoplasty, provides a new strategy for patients. Cornea repair and regeneration using stem cells has shown encouraging results in recent years. The key techniques depend on two aspects: one being the choice of the “seed cells” and the other being their carrier providing anchoring sites and structural support [93]. A recent literature review concluded that the corneal stromal stem cells, corneal limbal stem cells, embryonic stem cells, and other adult stem cells, as well as induced pluripotent stem cells, have been used as the “seed cells” [94]. In addition, the cell-free therapies such as EVs have also been explored for corneal regeneration [95]. Widyaningrum et al. [96] found that the platelet extracellular vesicles (PEVs) could increase the viability, wound-healing rate, as well as the proliferation markers of corneal endothelial cells, which represents a novel regenerative biotherapy for corneal endothelial dysfunction. The healthy human exosomes were also found to positively regulate the migration of human corneal stromal cell and wound healing in a recent study by Escandon et al. [97]. The studies summarized above clearly support the tremendous potential of EVs for regenerative medicine applications; however, further investigations in animals still need to be carried out for clinical translation. The human amniotic membrane is considered an ideal carrier for limbal stem cells (LSCs), which are often used for the cell-based therapy for ocular surface disease. However, Anton-Sales et al. [86] found that, compared with the human amniotic membrane, the BNC shows a slightly longer stability and higher mechanical resistance to sutures in vitro under physiological conditions, or ex vivo under simulated physiological conditions. Building on these findings, they attempt to utilize BNC as the carrier for human embryonic stem cell-derived LSC (hESC-LSC) to validate the transplant potential of these cells in a follow-up investigation [87]. The results showed that self-renewal and stemness characteristics of hESC-LSC can be maintained up to 21 days on the BNC substrates. Furthermore, the properties such as natural and accessibility of BNC might encourage more in-depth study for corneal regeneration.

### 3.2. Bioinspired Nanostructured Systems for the Treatment of Posterior Segment Ocular Disorders

As shown in Table 3, the research focused on the application of bioinspired nanocarrier for the treatment of posterior segment ocular disorders will be reviewed from the following four aspects: eye tumors, retina-targeting gene therapy, ocular neovascular disease, and other drug delivery system for posterior eye.

Uveal melanoma (UM) and retinoblastoma (Rb), which are threat to both vision and patient life, are the most common malignant intraocular tumors in adults and children, respectively [110]. For UM, radiotherapy and surgery has been the current first-line cancer treatment. However, radiotherapy may lead to vision impairment or loss due to a lack of tumor specificity. Furthermore, UM shows a high tendency for metastatic spread to the liver. In a retrospective study from 661 patients with a diagnosis of metastasis from UM, Lane et al. [111] found that the median survival time is only 3.9 months. The median survival time was 6.3 months for UM patients receiving treatment, compared with 1.7 months for those who did not receive treatment. Thus, early detection and treatment for patients with UM are essential to prolong survival. AU011, which is covalently linked the photosensitizer IRDye700DX (IR700) and can bind to the heparan sulfate proteoglycans (HSPGs) overexpressed on the surface of cell membrane through the capsid proteins produced by human papillomaviruses (HPV), is currently in the stage of clinical therapy for small primary choroidal melanoma including UM (ClinicalTrials.gov: NCT03052127). A study designed by Kines et al. [98] showed that AU-011 elicited significant and specific antitumor activity in a rabbit xenograft model of UM without off-target effects. This microbe-inspired nanoparticle may provide a novel first-line therapy for early-stage UM. For Rb, intra-arterial chemotherapy in one of the most commonly used therapy modalities. However, Rb continues to be a life-threatening ocular disorder due to its recurrence. The existence of cancer stem cells (CSCs) is considered one of the factors in triggering the recurrence of Rb [99]. The lactoferrin (Tf) receptor is over-expressed in the fast-growing tumor cells. Inspired by this, Narayana et al. [99] employed Tf as a nanocarrier to encapsulate carboplatin and etoposide for targeting CSCs in Rb cells. The study showed that the drug uptake and cytotoxicity of carboplatin- and etoposide-loaded Lf protein nanoparticles for Rb in the Y79 CSC population is significantly increased compared to the free drugs. However, as mentioned by the authors, deeper and more meticulous investigations in vivo in Rb CSC xenograft models are still needed to better realize the clinical translation. Inspired by the acid tumor microenvironments, the C-X-C chemokine receptor 4 (CXCR4) is overexpressed in Rb, and the elevated concentration of cellular glutathione, the CXCR4 antagonist (AMD11070) and DOX loaded, glycol chitosan-coated ceria nanoparticles (GCCNPs) were constructed by Gao et al. to treat the Rb [112]. Under physiological conditions, the AMD11070 and DOX are mainly located inside the GCCNPs. In an acidic microenvironment, the AMD11070 is exposed and binds to CXCR4 to realize specific targeting. After internalization, the bisulfide bond between DOX and nanoparticles will be broken under elevated concentration of cellular glutathione, leading to a selective drug release in Rb cancer cells. Compared to the DOX conjugated glycol chitosan (GCD), the GCCNPs could significantly enhance the anti-tumor activities of DOX in xenograft Rb mouse models. This “three-in-one” model provides a new combinatorial strategy for the preparation of bioinspired nanostructured systems for the therapy of Rb or other ocular tumors.

Inherited retinal diseases (IRDs), which arise from the pathogenic variants of 277 genes, is a clinically heterogeneous and complex group of visual lesions with phenotypes that affect retina function and exhibit a reduction in visual acuity [113]. Benefitting from current techniques for gene delivery, a new era of retina-targeting gene therapy for these vision threatening diseases has begun. The retina-targeting gene therapy involves the transfer of genes to target cells through subretinal (SR) injection or IVI of the engineered viral vectors. In recent years, adeno-associated virus (AAV), which can transfer genes to the retinal pigment epithelium, photoreceptors, as well as ganglion cells, has showed an increasing success in the therapy of IRDs in both pre-clinical and clinical trials. The retinal pigment epithelium-specific 65 kDa protein (RPE65) gene is one of the many mutated genes in leber’s congenital amaurosis (LCA). A follow-on phase 1 trial by Bennett et al. [100] covering 11 patients with childhood-onset blindness caused by RPE65 mutations indicated that one dose of recombinant AAV vector containing the RPE65 gene (AAV2-hRPE65v2) sub-retinally injected into to the contralateral eye in patients enrolled in the phase 1 study, still showed robust safety because of the immune privilege property of the eye. Furthermore, improvements in subjective and objective retinal and visual functions in patients were observed after 30 days of administration and a persistent improvement could be observed for at least 3 years (with observation ongoing). Hence, retina-targeting gene therapy using engineered viral vectors is a promising treatment option for IRDs as it can reduce dosing frequency and achieve long-term efficacy. Currently, SR injection is the main approach to deliver the treatment mediators to photoreceptors, which may be potentially harmful for the already compromised retina. Pavlou et al. [101] reported two novel engineered capsid variants called AAV2.GL and AAV2.NN, which were developed through a unique in vivo selection procedure in C57BL6/J mice. Via the single and relatively less invasive IVI route, the AAV2.GL and AAV2.NN could efficiently transduce photoreceptors in the *Cnga3*^−/−^ mouse model of achromatopsia and restored cone photoreceptor function. This study may revolutionize the therapeutic effect and accessibility of gene therapy for acquired retinal dystrophies or IRDs. Recently, the engineered AAV was also used to selectively upregulate the expression of silent information regulator 1 (SIRT1) in retinal ganglion cells of mice with experimental autoimmune encephalomyelitis (EAE) to increase neuronal cell survival and alleviate axon demyelination associated with optic neuritis [102]. The above data suggests a wide applicability and utility of gene therapy based on the recombinant AAV vector for retinal disorders occurring in the retinal pigment epithelium, photoreceptors, or ganglion cells.

Ocular neovascular disease can be divided into retinal vascular diseases and subretinal neovascularization, in which an increasing number of bioinspired nanocarriers have been developed and applied to treat choroidal neovascularization (CNV) and retinal neovascularization (RNV). AMD is characterized by CNV, while RNV plays a key role in diabetic retinopathy (DR). Because elevated levels of vascular endothelial growth factor (VEGF) binds to the VEGF receptor on the surface of endothelial cells in CNV and RNV, promoting neovascularization, specific targeting VEGF has been an effective method for the therapy of ocular neovascular disease [114]. Vascular endothelial growth factor A (VEGFA) is main representative isoform of VEGF. Lentiviral vector (LV) based clustered regularly interspaced short palindromic repeats/CRISPR-associated protein 9 (CRISPR/Cas9) genome editing and knockout system was constructed by Holmgaard et al. [104], which has shown a selective ablation of the Vegfa gene in in C57BL/6J mice through SR injection. They assumed that these findings may help develop effective treatment for ocular disorders including AMD. However, studies have shown the inflammation also promote ocular neovascular disease [115,116]. Hence, combination therapies of anti-inflammatory and anti-EGFR drugs may offer synergistic benefits to treat ocular neovascular disease. Inspired by the ability of regulatory T cells (Treg) to migrate to the site of inflammation and their anti-inflammatory potential, Tian et al. [103] engineered exosomes derived from Treg (rEXS), which were conjugated with anti-VEGF antibodies (aV) using a peptide linker to block VEGF activity with aV and mitigating inflammation. The results showed that the engineered exosomes could accumulate in CNV lesions and synergize rEXS-mediated immunosuppression, which was aV-mediated, to form a combination therapy in the laser-induced CNV mouse model through IVI. This combination strategy may benefit the development of treatments for other ocular neovascular diseases, including RNV. IVI of anti-VEGF is a main method for the therapy of ocular neovascular diseases. However, IVI, as we mentioned in the previous paragraph, has many side effects. Inspired by the ability of macrophage chemotaxis for inflammation and evasion of clearance by the reticuloendothelial system (RES), Xia et al. fabricated the macrophage membrane-derived vesicle (M-vesicle)-based nanoparticles and successfully delivered rapamycin to the CNV lesions in a laser-induced CNV mouse model through tail vein injection. Similarly, hybrid cell-membrane-cloaked nanoparticles from RBCs and the retinal endotheliocyte membrane were developed by Li et al. [106], and has realized the target and noninvasive anti-angiogenic therapy in laser-induced CNV mouse model after tail vein injection. Furthermore, based on the binding ability of vitamin B_12_ (VB_12_) to the intrinsic factors expressed on the surface of the intestinal lumen, Wang et al. [107] developed an VB_12_ modified oral nano-formulation and has enhanced the efficacy of scutellarin for the type II diabetes induced-retinopathy in a type II diabetic rat model. These studies shed new light on the noninvasive therapy for ocular neovascular disease.

In summary, many kinds of bioinspired nanoparticles have been developed to treat posterior segment ocular disorders. Encouragingly, other nanoparticles, such as the nanocarriers inspired by the oxidative stress in AMD [108] and the peptide transporter-1 expressed on the ocular surface [109], have been developed to deliver the therapeutic tools to the posterior segment. Since they have been reviewed in detail in Table 3, they will not be discussed further. However, it is reasonable to believe that the bioinspired nanoparticles could offer a safer, more effective, and non-invasive method for posterior segment ocular disorders.

## 4. Conclusions and Future Perspectives

After a systematic review of the existing literature, we recognized that significant progress in the therapy of ocular disorders has been achieved through bioinspired nanoscale drug delivery systems, especially microbe-inspired and animal-inspired drug delivery systems. Six core applications of the bioinspired nanocarriers for the ocular disorders were summarized here: ocular infections, regenerative ophthalmology, eye tumors, retina-targeting gene therapy, ocular neovascular disease, and other drug delivery systems to the posterior eye. For example, in the case of treatment of anterior segment ocular disorders, the bioinspired nanoscale drug delivery systems can address the problem of non-specific and uncontrolled drug release that existed in the traditional nano formulations. The bioinspired nanocarrier from abundant resources in nature provides more controllable, economical, and readily available materials for the regenerative ophthalmology. Furthermore, for the treatment of posterior segment ocular disorders, significant advances in bioinspired nanocarriers may bring the treatment of eye tumors, IRDs, and ocular neovascular disease into the era of precision medicine. Likewise, huge advances have been achieved by bioinspired nanocarriers in terms of the development of new minimally invasive or noninvasive administration routes to the posterior segment, including the IV, topical, and oral route.

Although the therapeutic potential of microbe-inspired and plant-inspired nanoscale drug delivery systems in treating different diseases has been investigated by many clinical trials, research of bioinspired nanocarriers for ocular disorders, besides the virus-inspired nanocarriers being tested in in phase 1/2 trials, is mainly limited to cells, animals, or ex vivo excised tissue experiments. Further investigation through reliable in vivo animal models is still needed before clinical trials. Benefitting from the immune privilege of the eye, the AAV2-hRPE65v2 used in a phase 1 study showed no inflammatory response in either eye and the immunological responses were benign. However, in some pathological situations, the leading contributor of immune privilege, including ocular resident cells/tissues to the privilege, will be affected. Hence, in-depth investigations and careful consideration of the micro-environment in different ocular disorders is required when establishing the nanocarriers. In turn, it may also offer new inspiration for the design of nanocarriers. Furthermore, the aforementioned exosome-like nano-vesicles formed in plants, which have been shown to largely avoid the immunogenicity, can be used to develop the delivery system of therapeutics or drugs for the ocular disorders. Presently, few plant-inspired nanoscale drug delivery systems have been used in ocular disease, while the natural components extracted from plants showed therapeutic properties. Carrier-free dual-drug nano formulations, which generally do not require any extra nanomaterials and are prepared by self-assembly of two drugs, can be utilized for the development of plant-based formulations. Many bioinspired nanoscale drug delivery systems are transiting from novel technologies to basic research; however, we believe that they have enormous potential for clinical translation with the in-depth awareness of ocular diseases and bioinspired nanoscale drug delivery systems.

## Figures and Tables

**Table 1:** The application of bacteria in different nano-drug delivery systems.

Bacterial Strain	Coating	Modifications	Therapeutic Agents	Study Goals	Ref.
*S. typhimurium* VNP20009	Poly(lactic-co-glycolic acid) nanoparticles	Streptavidin-biotin interaction	Immunotherapy	4T1 mammary tumors	[23]
*S. typhimurium* VNP20009	Polyamidoamine dendrimer	Electrostatic interactions	Immunotherapy	4T1 or CT26 mammary tumors	[24]
*S. typhimurium* VNP20009	Polydopamine	Electrostatic interactions	Immunotherapy	Melanoma	[25]
*S. typhimurium* Strain YB1	Indocyanine green-loaded nanoparticles	Covalent chemical conjugation	Photothermal therapy	MB49 bladder cancer	[26]
*Typhimurium* strain YS1646	Low-temperature sensitive Liposomes	Streptavidin-biotin interaction	Chemo-immunotherapy	C26 colon tumor model	[27]
*E. Coli* strain Seattle 1946	Mesoporous silica nanoparticles	Covalent chemical conjugation	Chemotherapy	HT1080 human fibrosarcoma cells in a 3D tumoral matrix model	[28]
*Clostridium novyi*-NT	Photosensitizer coated core/shell-structured lanthanide-doped nanoscintillators	Emulsification	Bacteriolytic and photodynamic therapy	PC3 prostate cancer tumor model	[29]
*Bifidobacterium* *bifidum*	-	Incubation and washing processes	Photothermal cancer immunotheranostics	Colon26-bearing immunocompetent mice	[30]

**Table 2:** The applications of bioinspired nanocarrier for anterior segment ocular disorders.

Disease	Inspirations from Nature Material or Functionals	Kingdom	Nanocomposites	Administration	Application Models
Ocular infections	Acid microenvironment in a bacterial infection lesion or biofilm	Microbe	EtNBSC nanoassemblies	IVI	Ocular bacterial infections in rats [80]
	Acid microenvironment in a bacterial infection lesion or biofilm	Microbe	ZIF-8-PAA-MB@AgNPs@Van-PEG	IVI	Mice endophthalmitis models [81]
	anterior ocular tissues in particular receptors, transporters, and GAGs	Human	CorTS 1 nanoparticles	Ex vivo corneas issues	Freshly excised goat eyes [82]
	TLR4 on the corneal epithelial cells	Human	Anti-TLR4 antibodies conjugated, ketoconazole-encapsulated gelatin nanoparticles	Topical route	Rat model of keratitis [83]
	Temperature of eye tissues	Human	PA and levofloxacin-loaded thermosensitive chitosan/gelatin-based hydrogel nanoparticles	Ex vivo t corneas issues	Rabbit model of *Staphylococcus aureus* keratitis [84]
	Temperature of eye tissues	Human	Thermosensitive gel containing sertaconazole-loaded NLCs	Ex vivo t corneas issues	Potential treatment of fungal keratitis [85]
Regenerative ophthalmology	BNC	Microbe	BNC hydrogels	Surgical suture	BNC hydrogels were sutured to pig eyes suture [86]
	BNC	Microbe	hESC-LSC loaded BNC	Surgical suture	Potential application on ocular surface regeneration [87]

Abbreviations: IVI, intravitreal injection; EtNBSC, amine group caged by an acid-cleavable moiety; ZIF-8-PAA, zeolitic imidazolate framework-8-polyacrylic acid; MB, methylbenzene blue; AgNPs, silver NPs; Van, vancomycin; PEG, polyethylene glycol; CorTS 1, corneal targeting sequence 1; GAGs, glycosaminoglycans; TLR4 Toll-like receptors; PA, prednisolone acetate; NLCs, nanostructured lipid carriers; hESC, human embryonic stem cells; LSCs, limbal stem cells; BNC, bacterial nanocellulose.

**Table 3:** The applications of bioinspired nanocarrier for posterior segment ocular disorders.

Disease	Inspirations from Nature Material or Functionals	Kingdom	Nanocomposites	Administration	Application Models
Eye tumors	VLPs	Microbe	Phthalocyanine photosensitizer conjugated VLPs	IVI	Uveal melanoma model in rabbit [98]
	Lf	Human	Carboplatin- and etoposide-loaded Lf protein nanoparticles	In vitro cell culture	Rb Y79 CSCs [99]
Retina-targeting gene therapy	AAV2	Microbe	AAV2-hRPE65v2	SR	Childhood-onset blindness caused by RPE65 mutations [100]
	AAV2	Microbe	Engineered capsid variants	IVI	*Cnga3*^−/−^ mouse model of achromatopsia [101]
	AAV7	Microbe	AAV7m8.SNCG. SIRT1	IVI	Mice with EAE [102]
Ocular neovascular disease	Exosomes derived from regulatory T cells	Human	VEGF antibody conjugated-exosomes	IVI	CNV mouse model [103]
	LVs	Microbe	CRISPR/Cas9 loaded lentiviral vectors	SR	Healthy mice [104]
	Membrane derived from macrophages	Human	MRaNPs	IV	Laser-induced CNV mouse model [105]
	Cell-membrane fusion by RBC and REC membrane	Human	Hybrid cell-membrane-cloaked PLGA nanoparticles	IV	Laser-induced CNV mouse model [106]
	IF on the receptors located in the luminal surface of the intestine	Human	VB_12_ modified, scutellarin loaded amphiphilic chitosan derivatives	Oral delivery	Type II diabetes-induced retinopathy [107]
Other drug delivery to the posterior segment	Oxidative stress in AMD	Human	Diselenide containing liposome	In vitro cell culture	hESC-RPE cells [108]
	PepT-1 on the ocular surface	Human	Multifunctional carboxymethyl chitosan derivatives-layered double hydroxide hybrid nanocomposites	Topical route	Healthy rabbits [109]

Abbreviations: VLP, virus-like particle; IVI, intravitreal injection; Lf, lactoferrin; CSC, cancer stem cell; Rb, retinoblastoma; LVs, lentiviral vectors; CRISPR, clustered regularly interspaced short palindromic repeats; SR, subretinal; AAV, adeno-associated virus; RPE, retinal pigment epithelium; SNCG, selectively in retinal ganglion cells under the control of the gamma-synuclein; SIRT1, silent information regulator 1; EAE, experimental autoimmune encephalomyelitis; Coga3, cyclic nucleotide gated channel subunit alpha 3; VLP, virus-like particle; VEGF, vascular endothelial growth factor; CNV, choroidal neovascularization; IF, intrinsic factor; VB12, vitamin B12; AMD, age-related macular degeneration; MRaNPs, rapamycin-loaded nanoparticles coated cell membrane derived from macrophages; RBC, red blood cell; REC, retinal endotheliocyte; RNV, retinal neovascularization; PLGA, poly-(lactic-co-glycolic acid); IV, intravenous; PepT-1, peptide transporter-1; hESC-RPE, human embryonic stem cell-derived retinal pigment epithelial.

## Data Availability

Data sharing not applicable.
